# Innovative Applications Enabled by the Versatile Structural Color of Cholesteric Liquid Crystals

**DOI:** 10.1021/accountsmr.5c00177

**Published:** 2026-02-25

**Authors:** Jan P. F. Lagerwall

**Affiliations:** Department of Physics & Materials Science, 81872 University of Luxembourg, 1511 Luxembourg City, Luxembourg

## Abstract

*Conspectus.* Cholesteric liquid crystals (CLCs) are famous for their ability to self-assemble into Bragg reflectors of visible light, yielding intense structural color with a single circular polarization, despite flowing like a liquid. This review focuses on a selection of entirely new opportunities to apply CLCs to solve problems with high societal and industrial relevance, as demonstrated in proof-of-concept experiments with a transition to commercial application underway, in contexts quite far from the more traditional applied role of CLCs as thermometers. We now see a renaissance of applied CLC research resulting in exciting new functional materials taking advantage of CLC photonics, often displaying unique types of responsiveness. This development has been enabled, first, by recent advances in formulating CLC mixtures with reactive mesogens such that they can be processed as a liquid but used as a hard glass or rubber after polymerization and cross-linking, keeping the photonic performance generated by CLC self-assembly intact. Second, the rapid development of advanced liquid processing methods like microfluidic production of multiple emulsions, 3D printing and composite fiber spinning have allowed the CLCs to be processed into unconventional form factors prior to cross-linking.

The review focuses, first, on CLC-templated hard spheres exhibiting omnidirectional circularly polarized Bragg reflection, so-called Cholesteric Spherical Reflectors, or CSRs. They can be used to make artificial “fingerprints” for physical objects that act as Physical Unclonable Functions, of great interest in secure authentication, or to print QR-codes or similar machine-readable patterns in a way that they remain invisible to humans while appearing to the intended machines with exceptional contrast. Since each CSR is effectively a pixel of structural color, we can also use them as a versatile solution for coloring without absorption or scattering, also enabling nonspectral colors like shades of gray that are normally not obtainable with structural color. A related application discussed is the camouflage of solar panels using polymerized CLC films to replace their visually obtrusive black appearance with color generated by CLCs, with almost no loss of energy conversion efficiency thanks to its origin in Bragg reflection.

We then move to soft rubbery CLC elastomer (CLCE) films and fibers which change their color in response to strain. We highlight a new application opportunity in structural health monitoring, demonstrated by coating CLCE films onto surfaces where we wish to detect crack formation, e.g., in reinforced concrete constructions: the localized strain in the CLCE where a crack appears leads to a strong color change that allows immediate detection of the crack, whereas the crack in the uncoated surface remains invisible until it has grown to much greater width. The colorimetric strain monitoring is also possible with CLCE fibers, where the 1D form factor lends itself to applications in, e.g., fashion, medicine and sports. We end by discussing the key remaining challenges, in particular related to scale-up of production.

## Graphical abstract

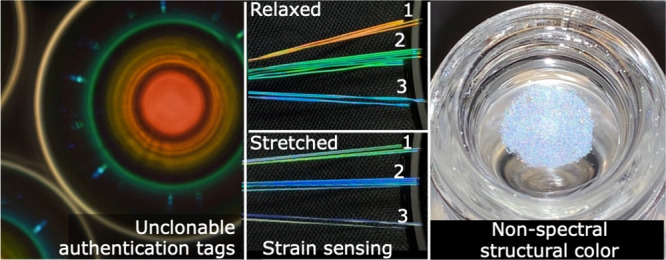

## Introduction

Could a 137-year old material still have anything new to offer in the fast-paced age that we we live in? For the case of Friedrich Reinitzer’s 1888 discovery of cholesteric liquid crystals (CLCs)—which was also the discovery of liquid crystals (LCs) in general^1^—this is definitely the case. The realization that a liquid crystalline state of matter exists was itself so disruptive that a whole new body of physics, chemistry and engineering had to be developed, and the resulting challenges and possibilities have fascinated generations of scientists. The unique combination of liquid-like mobility with long-range orientational order gives rise to a plethora of intriguing phenomena in LCs. For this article, the foundation is the ability of CLCs to generate flowing shape-shifting Bragg reflectors creating beautiful structural colors and highly complex optics. This is the result of the CLC *mesogens*—the molecules, polymers or nanoparticles that generate the phase—self-assembling with long-range orientational order along a *director* **n**, which, in turn, twists around an axis **m**⊥**n** with a period, or *pitch*, *p*. The long-range orientational order leads to optical anisotropy and the twisting yields a periodic modulation of the effective refractive index, which, since *p* is often on the order of some 100 nm, gives rise to Bragg scattering of visible or near-visible light. Importantly, the Bragg-reflected light is circularly polarized with the same handedness as the CLC twist.

I will attempt to illustrate the many modern-day innovative application opportunities that arise when combining a solid understanding of CLC physics and the most recent innovations in LC chemistry with an open mindset that enables connection with relevant technical and business challenges. Among the reasons why CLCs attract such strong attention these days is, first, that cholesteric chemistry has diversified on a grand scale over the last decades, such that there is now a rich palette of mesogens, each with their unique advantages. We have a whole menu of reactive mesogens that can be polymerized into either solid glasses or soft elastomeric networks while maintaining the LC-derived order.^2^ Furthermore, we have multiple powerful chiral dopants that enable convenient and continuous tuning of *p*, and thus of the characteristic cholesteric reflection color, from the infrared (IR), throughout the visible spectrum, into the ultraviolet (UV). Finally, the traditional bottom-up-synthesized petroleum-based mesogens are complemented by bioderived polymeric cholesteric phase generators,^3–5^ for instance hydroxypropylcellulose forming colorful aqueous solutions or cellulose or chitin nanocrystals (CNCs and ChNCs, respectively) that can be suspended to form cholesteric phases, with strong colors arising upon removal of the water.

A second reason is that several societal and industrial challenges have arisen in the last years to which powerful solutions may be found by harnessing the versatile structural color of cholesterics, from addressing the surge of counterfeiting^6^ or providing machine-readable information without filling our environments with visibly disruptive QR-code-like markers,^7^ to the urgent need for renewable energy solutions that blend seamlessly into our landscapes.^8^ With this review, I hope to show how these developments, where an increasingly versatile CLC platform meets novel global challenges in which cholesteric optics can find innovative uses, combine in a thriving new research climate for the field. The key distinction from other recent reviews of CLCs, by other research teams^4,9–12^ or by my group and collaborators,^13,14^ is a sharp focus on a few selected applications—unorthodox and spanning a very broad range of contexts, yet already demonstrated in realistic scenarios to have potential to solve societally and economically important problems—of CLCs polymerized into solids or elastomers. Focusing entirely on the applications, this review assumes basic acquaintance with CLCs and their optics. For readers who are new to this field, good introductions may be found in the mentioned prior review articles.

### Secure Authentication Based on Cholesteric Spherical Reflectors (CSRs)

As supply chains have become increasingly globalized, the number of opportunities for fraud, forgery, or outright sabotage has dramatically increased, and the current situation poses a serious threat.^6^ Original components may be diverted and sold on the black market while fake or substandard components replace them in the continued supply chain. Technologies that can provide secure authentication of physical items are thus urgently needed. CLCs molded into spheres with radial **m** and then polymerized into glassy solids, what we refer to as cholesteric spherical reflectors (CSRs), can provide a very interesting solution. This is due to their rich optical response, including omnidirectional selective Bragg retroreflection,^14^ their beneficial balance between control and randomness, and the wide opportunities for tuning the controlled features of CSRs, such as *p*, twist handedness, diameter, etc. Following a carefully designed microfluidic process, CSR shells with excellent optical quality can be reliably produced,^15^ and if hybrid boundary conditions are applied even more complex optics are achieved.^16^ By depositing a binder with a random mixture of different types of CSR onto a surface and then solidifying the binder we create a “fingerprint” that can be probed by analyzing the CSR response to different illumination conditions.^17–19^ Because the CSR locations and orientations are random, each fingerprint is unique, and because a variation of the illumination conditions continuously changes the response, there is an infinite set of challenge–response functions. This turns the CSR array into a physical unclonable function (PUF), a highly desirable material in the domain of secure authentication.^20^

For more than a decade now, I have been working with computer scientist Prof. Gabriele Lenzini, an expert in cyber security and secure authentication, in analyzing the optical patterns generated by CSR fingerprints and converting them robustly into digital representations.^17–19,22^ With the algorithms developed by his team,^21,23^ the PUF characteristics of a certain fingerprint can be analyzed using low-cost digital equipment (Figure 1a) and stored in a database, such that the object carrying the fingerprint can have its authenticity confirmed through a set of optical tests and comparing the response with that in the database (Figure 1b). Gabriele and I recently started the company Trace Crystal, together with two former postdocs, Dr. Hakam Agha and Dr. Mónica Arenas, which is now developing the concept into a product. The company offers authentication of jewelry and artworks and it will soon offer a version that is adapted to the needs of high-volume products suffering from counterfeiting and return fraud, in particular pharmaceuticals.

**Figure 1:**
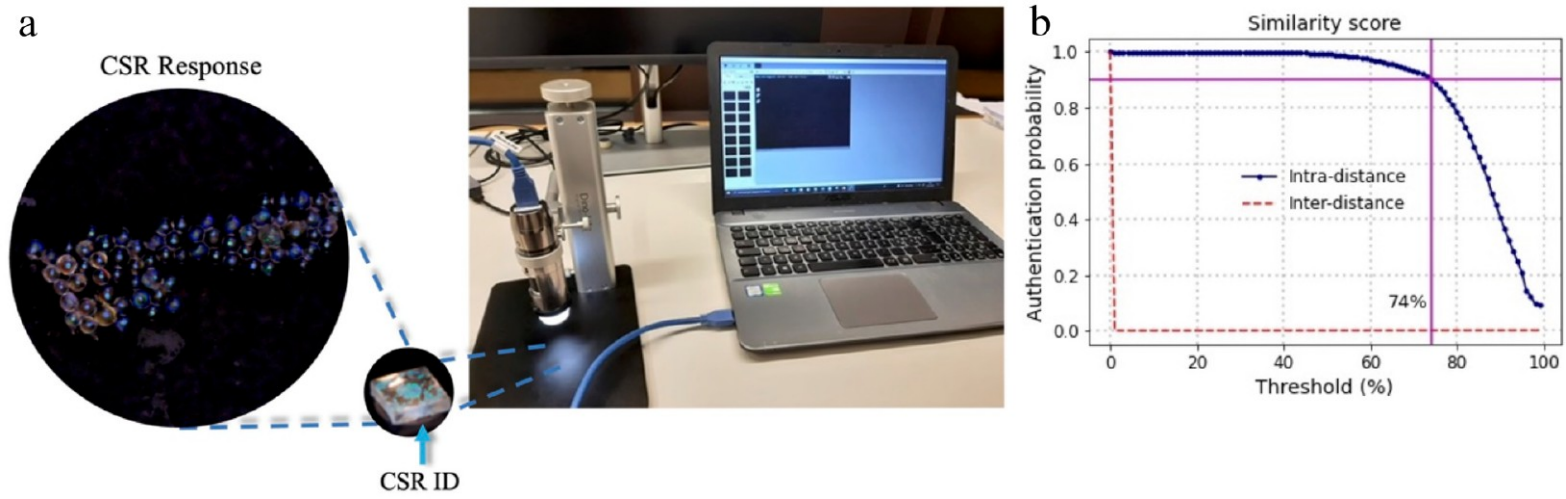
(a) The optical response of a CSR fingerprint (CSR ID; ∼2 mm radius), is recorded by a microscope connected to a laptop running the authentication software. (b) True/false-positive acceptance rate at changing similarity score between enrolled pattern and rereading. A threshold of 74% gives a true acceptance rate of ∼90%. [Adapted with permission (CC-BY license) from ref 21. Published by MDPI, 2022.]

### Human-Invisible Information Encoding Using CSR Optics

Over the last years, we have all become used to scanning QR codes, the most well-known example of information encoded into graphical patterns designed for reading solely by machines. When we point our mobile phones to a QR code, we are using the simplest form of Augmented Reality (AR), where the physical world is augmented by information relevant specifically for the item carrying the code, provided in text, video, or audio via our phone. Some less well-known relatives are fiducial markers and data matrix labels (see Figure 2). Like QR codes, they have a square footprint and encompass a set of black lines and squares on a white background. Data matrix labels are common on electronics or pharmaceuticals where space is limited, because QR codes require larger size for reliable reading. Fiducial markers are artificial landmarks that allow the machine reading the markers to identify where in the field of view objects are located and how they are oriented.^7^

**Figure 2:**
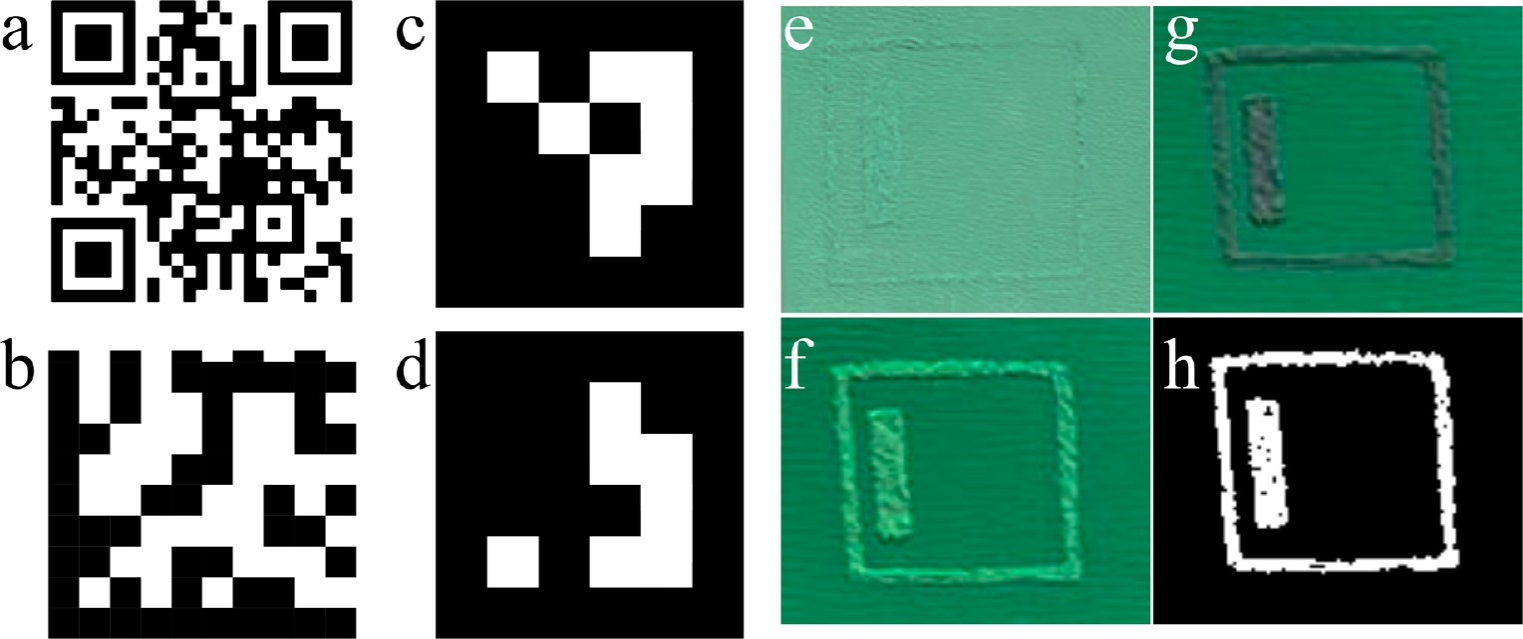
Graphically encoded information for reading by digital devices. (a) A QR code pointing to the author’s group’s Web site. (b) A data matrix representing the letters ”CSR”. (c, d) Fiducial markers from the ArUcO library representing 0 and 1. (e) Left-handed green CSRs forming a fiducial marker-like pattern on a green background. (f) Viewing through a left-handed circular polarizer the pattern is more visible since the background intensity is reduced by 50% while the CSR reflections remain unchanged. (g) The contrast is inverted if viewing through a right-handed polarizer. (h) Subtracting panel (g) from panel (f) and switching to monochrome, the marker pattern appears with exceptional contrast. Panels (e)–(h) have been used with permission from the Ph.D. research of Dr. Deniz Işınsu Avşar .

Robots and AR devices that need to navigate in a complex space are greatly aided by fiducial markers placed on doors, walls, mobile objects etc., as they allow robust object identification and localization. Doing the same without markers using artificial intelligence on a regular video feed requires much greater computational power (and thus energy consumption), radio bandwidth, and time. To be useful, the markers need to appear to the machine with strong contrast and with sufficient size. In their current form, they thus constitute significant visual pollution, which greatly limits their useage. You may see fiducial markers at super markets, airports, hospitals or other busy places, where they assist cleaning or inventory management robots in their navigation.

I first learned about fiducial markers in 2013 from Prof. Mathew Schwartz, an architect at the forefront of using robots in construction of the built environment. Together, we realized that the omnidirectional selective retroreflectivity of CSRs offers a very interesting route to deploying graphical markers to a much greater extent, a concept that we first published together with Gabriele and his team in 2018.^18^ First, we make the markers using CSRs reflecting light outside the visible spectrum or with a color that matches the background, such that humans do not notice them. Second, we use the circularly polarized CSR reflection to ensure that the device sees *only* the markers, nothing else. Let me show you how it works.

The key is to obtain two images of the same scene, one recorded through a right-handed circular polarizer and the other through a left-handed circular polarizer, and then subtract one from the other. Because practically nothing in the natural world is circularly polarized, the only difference between the two images are the CSRs. Thanks to their spherical shape, their appearance is largely viewing angle-indepedent, in contrast to that of flat CLCs.^14^ Assuming we use left-handed CSRs, a marker made with them will appear bright in the left-handed but dark in the right-handed image, while everything else appears identical (see Figure 2f and 2g). If we subtract the right-handed from the left-handed image, only the marker remains (Figure 2h), which allows it to be detected with unprecedented clarity. Figure 3 shows how this subtraction can be done in real time using a simple dual-camera setup developed within a fruitful collaboration with the robotics engineering group of Prof. Holger Voos.

**Figure 3:**
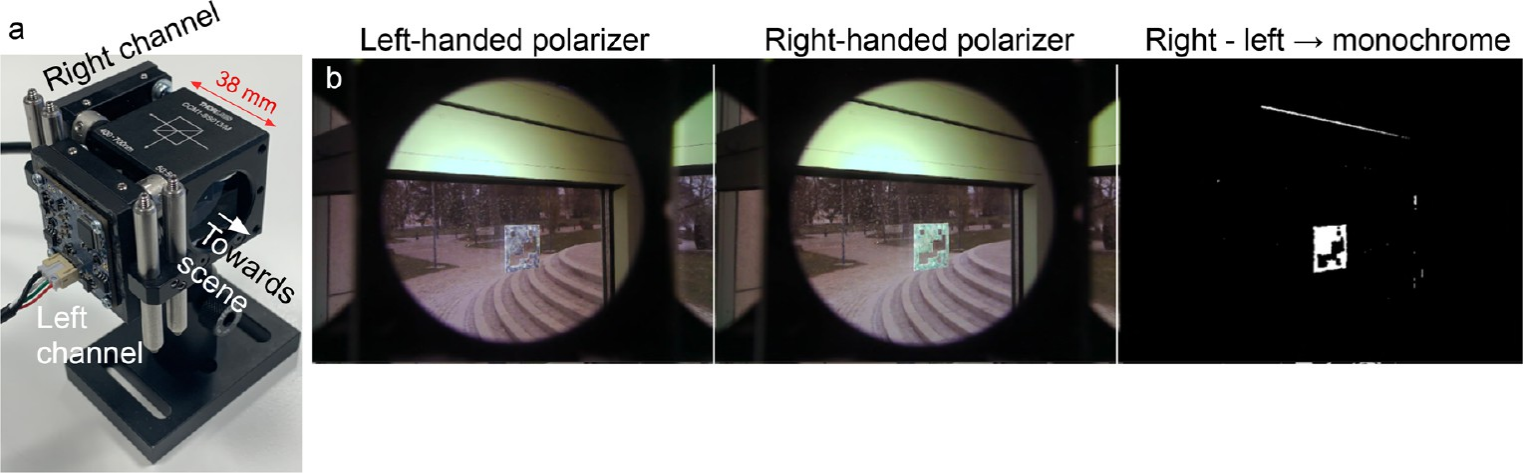
(a) A dual camera + circular polarizer + beam splitter setup for parallel imaging CSR detection and realtime background subtraction. (b) The output from the software that reads and processes the data from the two cameras. Here, a fiducial marker made using right-handed green-reflecting CSRs is mounted on a window. The device was designed and manufactured by Dr. Hakam Agha and the software was written by Mr. Ali Tourani. [Adapted with permission (CC-BY license) from ref 14. Published by Springer Nature, 2022.]

For ensuring that the human eye cannot detect the CSR reflection, the most efficient is to move the retroreflection wavelength λ_0_ to just beyond the short-wavelength edge of the visible spectrum, e.g., λ_0_ ≈ 380 nm, see Figure 4. The advantage of working with such near-UV CSRs is that all non-retroreflections, i.e., Bragg scattering under oblique light incidence, occur even further into the UV range, thus they are also invisible to the human eye. Another option is to camouflage the CSRs by designing them to reflect in the visible range, and ensure that their effective color matches that of the background, such that the human eye does not notice the markers for this reason. A good example is shown in Figure 2e. This brings us to another very interesting aspect of CSRs, namely, the possibility of using them as alternatives to conventional pigments for creating color.

**Figure 4:**
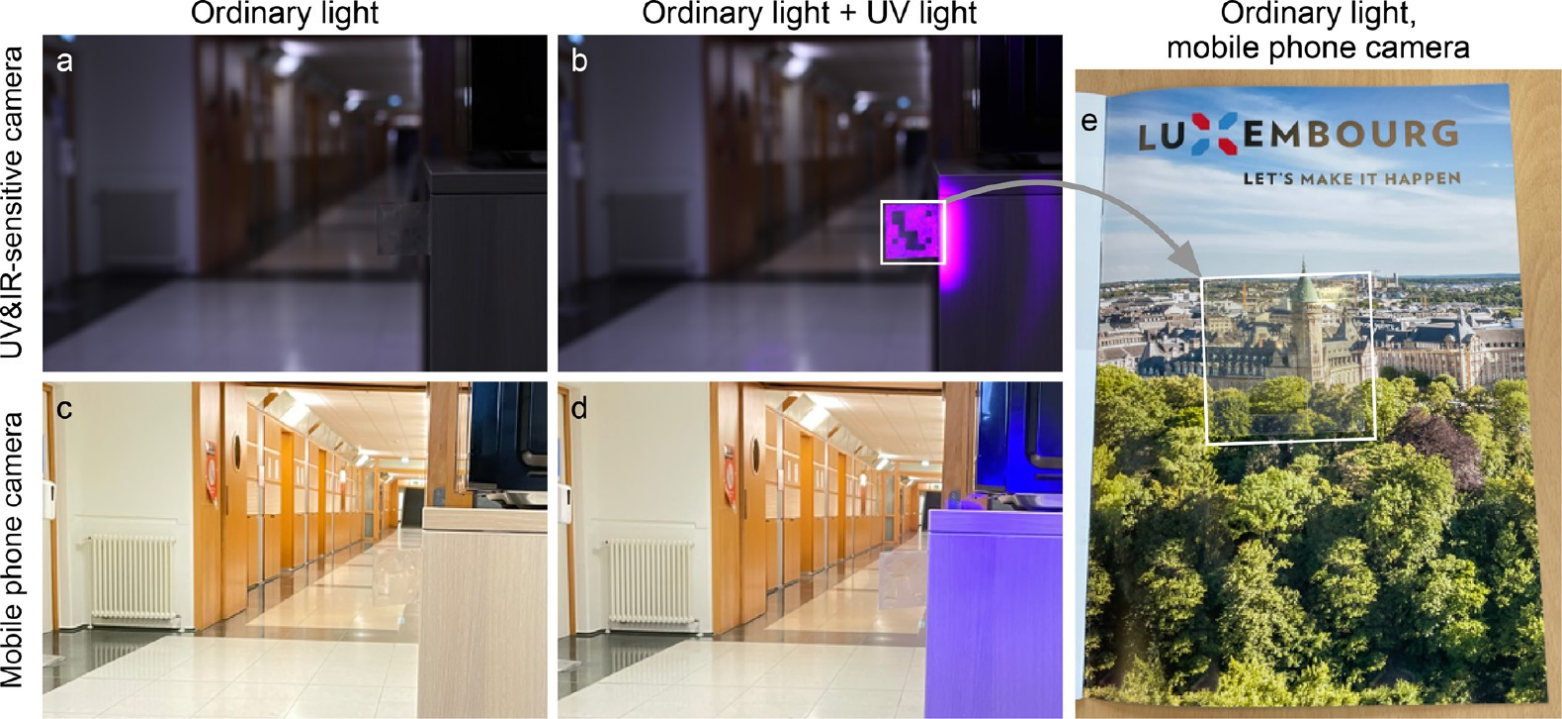
A marker made using near-UV reflecting CSRs embedded in NOA glue, imaged by a UV-sensitive camera (a) without and (b) with UV irradition turned on. When a regular camera is used, which features UV- and IR-blocking filters, it is difficult to spot the marker (see panels (c) and (d)). The same marker is placed over a brochure and it is so difficult to see that we highlighted it by a white frame (panel (e)). [Adapted with permission (CC-BY license) from ref 14. Published by Springer Nature.]

### Versatile Structural Color Using CSRs

Structural color is a very interesting approach to coloration. If it is generated using bioderived materials, like cellulose- or chitin-derived cholesteric structures, great environmental gains can be achieved.^24–27^ The fact that color is generated without absorption is hugely beneficial in light harvesting contexts, as illustrated in the next section. However, structural color has two significant drawbacks: the apparent color depends on illumination and viewing directions,^13,14^ and the selective reflection is limited to spectral colors, not including white, gray, pink, brown, and so on.

CSRs offer a means to address these drawbacks. First, the viewing angle dependence is greatly reduced thanks to the spherical shape and the omnidirectional retroreflection. Second, the discrete nature of CSRs effectively turns them into pixels of structural color that allow us to generate apparent nonspectral colors by color mixing, as in standard RGB displays. If we make CSRs that appear red (R), green (G), and blue (B), respectively, and mix them in varying proportions, we can generate almost any color.^28^ A random mixture of R-, G-, and B-CSRs suspended on a water surface gives an excellent gray–white appearance when viewed with the naked eye (Figure 5a–c), confirming the feasibility.

**Figure 5:**
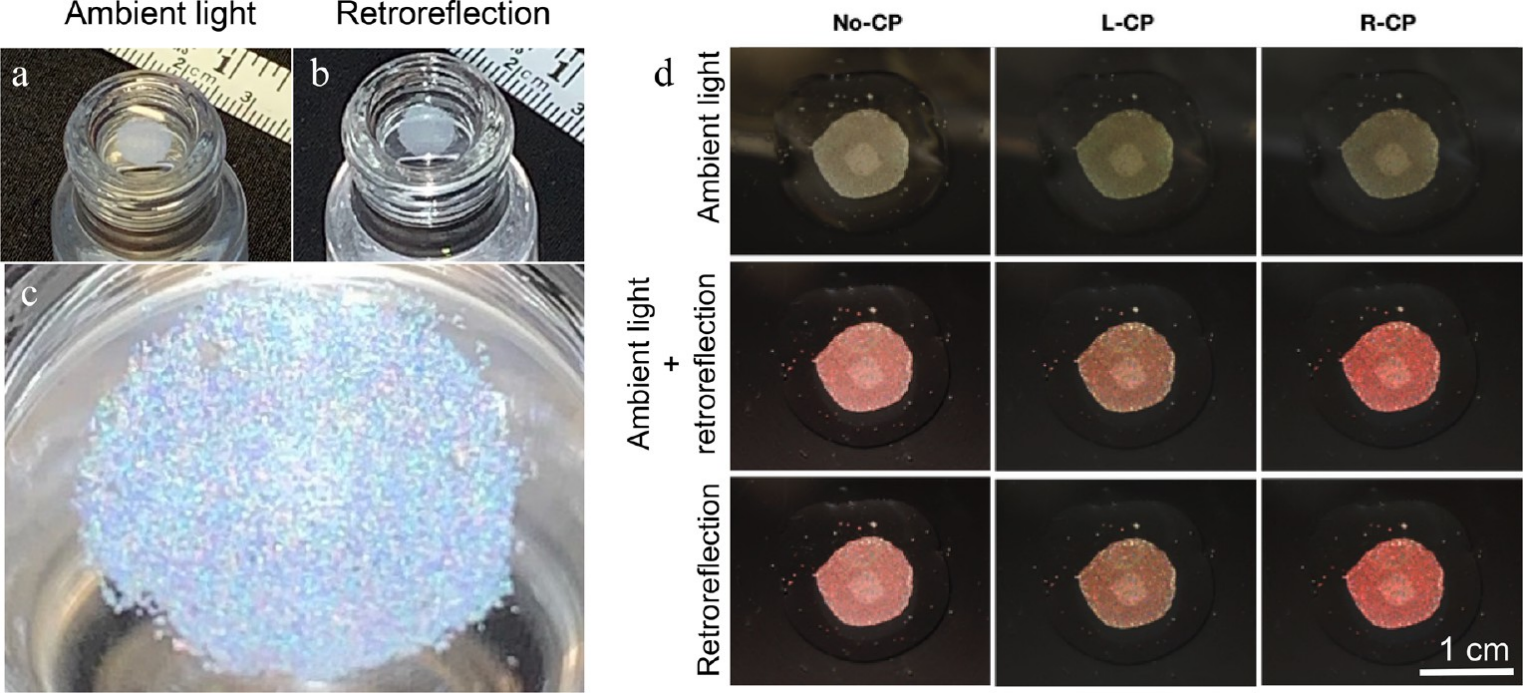
(a–b) CSRs as structural color “pixels”: mixing red, green and blue CSRs in equal proportions yields a white macroscopic appearance. (c) Zooming in, we can see the individual R-, G-, and B-pixels. (d) To obtain a film that appears red in retroreflection, we use CSRs with λ_0_ = 880 nm. Under ambient light (top row) the high-angle illumination components lead to a blue shift. [Adapted with permission (CC-BY license) from ref 28. Published by Wiley, 2023.]

However, the control of the apparent color is more complex than with conventional pigments or dyes. With oblique illumination, we get direct reflections at wavelengths shorter than λ_0_, and, even under retroreflection conditions, the effective color is nontrivial to predict because an array of CSRs reflects blue-shifted light back to the observer via a mechanism termed *photonic cross communication*,^29^ and CSR shells also feature a blue-shifted internal retroreflection mode.^30^ B-CSRs are largely insensitive to the issue, since their blueshifted reflections are mainly in the invisible UV-range, but G-CSRs and even more R-CSRs are strongly affected. The problem was noted also by Belmonte et al., who solved the problem by mixing dye into the CSRs, which absorbs the cross communication color.^31^ While this is indeed a very effective way of increasing color purity, it is not a solution when we wish to avoid light absorption in the color generation.

A benefit of the color mixing approach is that nonidentical CSRs in a plane strongly reduces the cross communication and thus diminishes the resulting color distortion, even without any dye. But if we want to make a red or green sheet we must work with identical CSRs, and we therefore explored the alternative to strategically red-shift λ_0_, with respect to the desired color. For instance, with λ_0_ ≈ 880 nm CSRs, the macroscopic appearance under retroreflection became red, thanks to the cross communication (see Figure 5d). Under ambient light, the same coating had a more greenish brown appearance. Our conclusion is that it is possible to generate a broad spectrum of color without absorptive elements using CSRs, but finding the right composition of CSRs is more complex than conventional RGB color mixing.

## Polymerizing Flat Films of Cholesteric Liquid Crystals

The same or related cholesteric precursor mixtures as used for CSRs can, of course, be molded into flat films or other shapes, such as cylindrical or ribbon-like fibers, and then polymerized. Each form factor has its own pros and cons. In this section, I will focus on flat films, discussing two particularly exciting applications in some detail: (i) giving solar cells color without ruining their efficiency, and (ii) structural health monitoring.

### Using Cholesterics To Make Highly Efficient Colored Solar Cells

With climate change giving more and more frequent reminders of its impact, it is clear that new energy solutions for the world are urgently needed. Harvesting the power of wind, water, and sun must clearly play an important part and the technologies are now so well developed that they can truly deliver, at a reasonable financial cost. The cost that is more difficult to accept for many people is that of the visual and audible impact and the physical footprint of the installations.^8^ Here, I wish to focus on photovoltaics (PV), where the main challenge is the visual pollution of large black panels being installed in the environment in which we live, work, and play.

A truly game changing development would be if the PV panels could be camouflaged and integrated in buildings such that people do not notice them. The PV industry is therefore working hard on Building–Integrated Photovoltaics (BIPV),^32^ where a key component is to make the panels visually attractive, preferably having any appearance a customer may desire. The simplest solution is to print pictures over the panels, yielding great results in terms of aesthetics, but since conventional dyes and pigments absorb and scatter light the cost in terms of reduced energy conversion efficiency is disastrous.

In many respects, PV technology and structural color is a match made in heaven, since structural color requires a black background for generating saturated color, and a standard PV panel is pitch black. Several companies thus offer PV panels with thin-film structural color coatings, maintaining good energy conversion. However, most such coatings are made in processes that are difficult if not impossible to apply on curved building elements, and they work only for a single color covering the entire panel, far from offering camouflage. In addition, the viewing angle problem means that the BIPV element will change appearance as the sun moves over the sky during the day and also as people walk by the element.

In a fruitful collaboration with the Photovoltaic Materials research group of Prof. Phillip Dale, we recently showed that polymerizable CLCs offers a highly attractive alternative.^33^ Because we start with a liquid precursor that self-assembles into a periodic structure, we can coat any shape of surface with the precursor, and because we can tune *p* both by chemical composition and temperature we can easily vary the color across the surface as we wish prior to polymerization. We demonstrated the possibilities using a polymerized cholesteric film patterned into a regular array of orange, green and blue pixels of about 1 mm^2^ area each (Figure 6a–d), giving the solar cell a nonspectral apparent color that blended in extremely well over textured building materials such as a wooden facade (see Figure 6e and 6f). To make this coating, Dr. Yansong Zhang formulated a reactive cholesteric mixture with strong temperature dependence of *p* and coated the solar cell with it. He heated the sandwich up to a temperature where the mixture reflects in the blue and then UV-cured those pixels that should be blue by irradiating through a photo mask. He then cooled it to a temperature where the mixture is green and repeated the process for the pixels that should be green. Finally, he cooled it to room temperature and UV-cured without any mask, since all remaining pixels were intended red. Since our mixture and procedures were not entirely optimized, the last pixels ended up orange, but the film actually blended in better onto wooden surfaces than a white color (see Figure 6e and 6f.)

**Figure 6:**
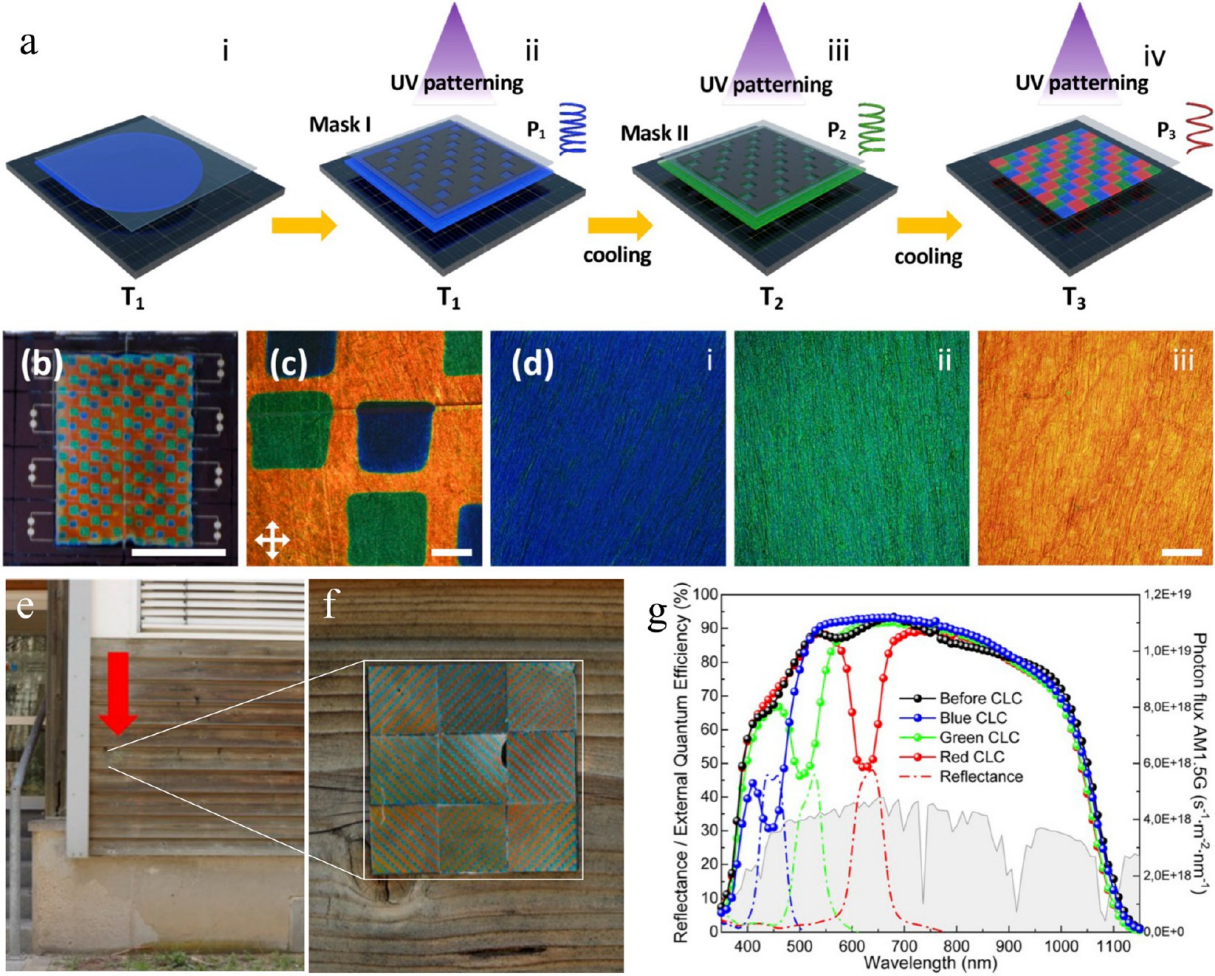
Camouflaging solar cells with minimum impact on photoconversion efficiency using polymerized CLC. (a) The scheme for photopatterning the reactive cholesteric at three different temperatures to achieve a grid of orange, green, and blue pixels. (b) A photo showing such a grid covering eight PV cells (scale bar: 1 cm). (c–d) Micrographs of the film and the respective areas (scale bars: 400 and 100 μm, respectively). (e, f) A 3 × 3 array of grids camouflaged against a wooden house wall. (g) Reflection spectra (dashed) and external quantum efficiency (EQE) spectra (continuous) of PV cells covered by uniformly red, green, and blue (in retroreflection) polymerized CLC, along with the representation of the AM1.5G photon flux (gray shaded area) plotted against the right axis. A reference EQE spectrum without LC is plotted in black. [Adapted with permission (CC-BY license) from ref 33. Published by Royal Society of Chemistry, 2025.]

The cholesteric-derived coating has one further advantage: even within the reflection band half of the incoming light reaches the solar cell, since only the circularly polarized component with the same handedness as the director twist is reflected. As is well-known from biological samples of cholesteric -derived structural color,^9^ the reflected light fully suffices to give very strong colors. As a result, we get excellent color on the solar cell, yet its energy conversion efficiency remains very high, as shown in Figure 6g. Even in the red pixels (largest reflection bandwidth) we have more than 90% retained efficiency. In a project designed to take our solution out of the lab and into the real world, our combined team is now working to bring this PV coloring technology to market in collaboration with commercial BIPV providers. Our timeline aims at company creation and first commercial products in 2027.

### Structural Health Monitoring Using Cholesteric Rubber Coatings

If we combine the typical diacrylate reactive mesogens with short flexible dithiol chain extenders, we can obtain cholesteric-templated volume-spanning networks that are elastomeric, or rubbery, in character. Such CLC elastomers (CLCEs) have spectacular mechanochromic properties, i.e., they change their reflection color in response to mechanical deformation.^34^ Dr. Rijeesh Kizhakidathazhath in our group developed a new chemistry route that makes it exceptionally simple to make CLCEs, and I am glad to see that his breakthrough has inspired much follow-up activity.^35–40^ The fundamental principle is straightforward (Figure 7a). Consider a flat film prepared with **m** normal to the film plane that is stretched in this plane. Since the CLCE is incompressible, the in-plane extension must be compensated by a decrease in the thickness. Since the twisted director field is locked into the rubbery structure, *p* decreases as much as the film thickness, hence the film experiences a blue shift that quantitatively reflects the mechanical deformation.

**Figure 7:**
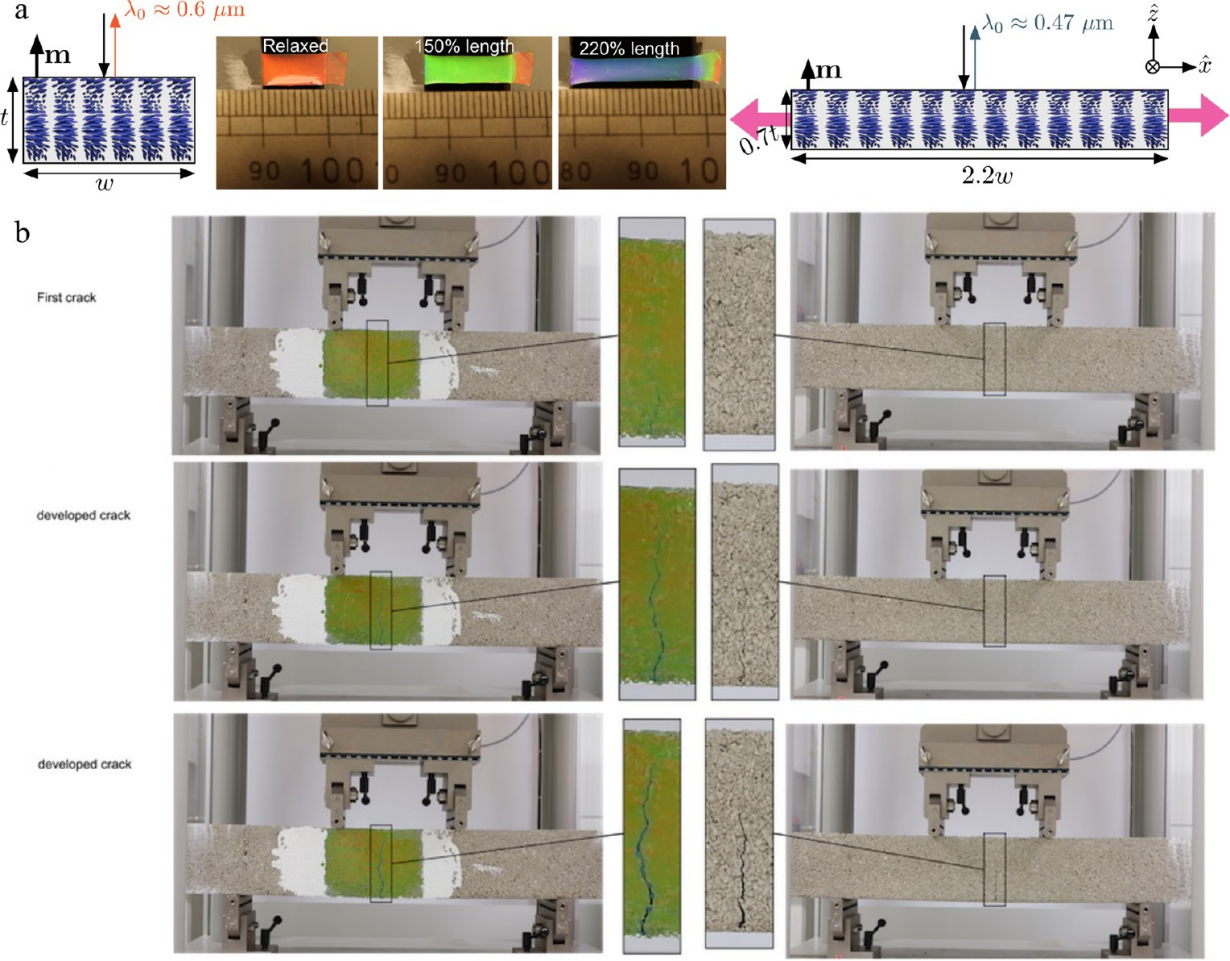
(a) Mechanochromic CLCE response illustrated with a film that is red in the ground state and blueshifts all the way to violet upon uniaxial stretching. (b) A CLCE coated onto a beam (113 cm long, 17.5 cm wide, 11.5 cm thick) of reinforced concrete subject to bending reveals crack formation immediately (left column, top row), whereas the crack in a beam without CLCE becomes visible only long after formation (right column, middle row). Adapted with permission (CC-BY license) from ref 41

There is a host of ways in which the mechanochromic CLCE response can be applied. Here, I will discuss only one that is particularly impactful, namely, as a functional coating of concrete, steel etc for structural health monitoring (SHM). I have been pursuing this topic in collaboration with Prof. Danièle Waldmann-Diederich, recently also with Prof. Numa Bertola, who are both experts in concrete engineering and SHM. Our built environment is aging, and to avoid tragic events like the Morandi bridge collapse in 2018, there is a strong trend today in the field of construction engineering to integrate systems for SHM. A key task is to detect cracks at a very early stage and monitor their further growth, such that action can be taken well before a catastrophic collapse takes place. Currently, SHM is still based largely on manual inspection, inefficient and infrequent. Moreover, by the time a crack is visible to the human eye the structural health may already be significantly deteriorated. Systems for automatic SHM often involve discrete optical or electronic crack monitors, all quite costly while being prone to miss cracks that develop away from the detector.

CLCEs are highly interesting for SHM if applied as coating onto a surface, where they function as fully continuous 2D crack detectors: no matter where a crack appears within the covered area and no matter in which direction it runs or bifurcates, the CLCE will reveal it by a local blue shift since the crack stretches the CLCE, as demonstrated in Figure 7b.^41^ Importantly, it is exceptionally easy to coat the target surface with the CLCE, because one can apply a liquid precursor including a volatile solvent either by brush^41^ or spray^38^ coating, and as the solvent evaporates the Michael addition polymerization completes.

For this application, a critical issue is cost. Once reactive cholesteric mesogen chemistry becomes mainstream, the production costs will go down, and as the processing of cellulose-based precursors develops we may expect an even greater price reduction. However, already with today’s petroleum-derived specialty chemicals, the cost is low, compared to competing technologies: we estimated it at 50 Euro per square meter. Also the equipment needed to monitor the CLCE response is inexpensive, typically based on standard color cameras and white LED light sources, possibly mounted on drones for covering a larger area.

In the context of CLC-based mechanochromism, it is worth mentioning another form that has already been on the market for more than a decade, where localized pressure is used to realign liquid-state, non-cross-linked, CLCs. This is the basis for the writing tablet Boogie Board, from Kent Displays. In the ground state, the CLC is aligned between plastic pillars with **m** in the board plane, such that no color is reflected. Pressure by the stylus induces localized flow that, guided by the pillars, realigns **m** perpendicular to the board plane below the stylus tip, giving a local green appearance.^42^ This binary (black to single-color) piezochromic response is nonelectronic and in the writing phase the board thus consumes no power. A battery is still needed because a brief electrical pulse is used to reset the board, bringing **m** back into the plane of the board to recover the original dark color.

## Fibers of Cholesteric Rubbers

I end this review with CLCE fibers, showing the same mechanochromic response as the films. Because the fibers can be sewn into a host fabric or—once long enough continuous threads are available—woven or knitted into a fabric entirely made out of CLCE, and as they are machine washable,^43^ many interesting applications can be envisioned in garments for sports, medicine, fashion and much more. There are two significant challenges in making CLCE fibers. First, the liquid precursor must be prevented from breaking up into droplets via the Rayleigh–Plateau instability. When making nonchiral nematic LCE fibers for artificial muscles this problem is solved by extruding in the nematic phase with immediate UV curing.^44–47^ This can be difficult to apply for CLCE fibers, because annealing prior to curing is normally required to get uniform and appropriate orientation of **m**. The configuration we want is one with radial **m** and to achieve this we have developed two alternative approaches.

First, we deposited an isotropic precursor solution of cholesteric LC oligomer (LCO) onto a rotating cylindrical mandrel such that it forms a spiral.^43^ With the right concentration of LCO, the solution is so viscous that the Rayleigh–Plateau instability does not break up the deposited filament and the cholesteric structure has time to develop as the solvent evaporates. Once it is annealed, the precursor filament is UV-cured and the CLCE fiber is harvested from the mandrel. While this procedure works well, the fiber ends up ribbon-shaped, see Figure 8a. The lack of cylindrical symmetry has some drawbacks, not least in case of sewing, weaving, knitting and knotting, since the warping of the thread will expose different sides of the ribbon to the viewer, with different optical impact.

**Figure 8:**
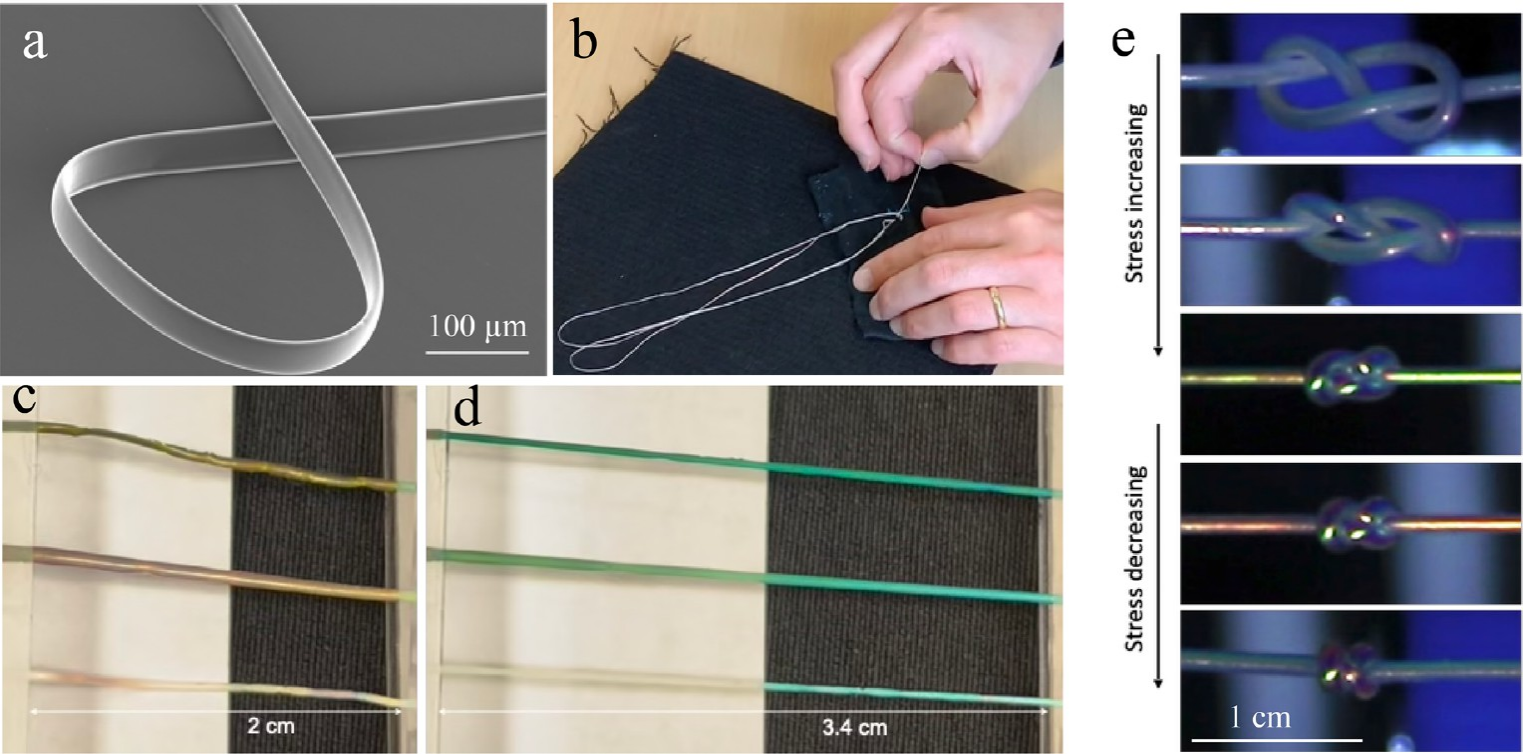
Mechanochromic CLCE fibers. (a) SEM image of a ribbon-shaped fiber made on a rotating mandrel. (b) Hand sewing of the CLCE fiber into a regular textile cloth. (c, d) The color change upon stretching is visible over white and black backgrounds because the CLCE has been infused with a black dye. (e) Strain monitoring in a cylindrical CLCE fiber made by tube templating as a knot is being tightened, and then the fiber is released. Panels (a)–(d) have been reproduced with permission (CC-BY license 2022) from ref 43 (Published by Springer Nature, copyright 2022) and panel (e) has been reproduced with permission (CC-BY license) from ref 48 (published by Wiley–VCH).

For this reason, our second solution is now our method of choice.^48^ In this case, we introduce the precursor, which can be an LCO solution or a monomeric mixture, into a tubular template. The precursor is left in the tube long enough for annealing with radial **m** and then the precursor is cross-linked into an LCE by UV-curing and the tube is dissolved. This yields excellent cylindrical fibers with full rotational symmetry about the fiber axis, which are ideal for textile applications. Figure 8e demonstrates an application in monitoring the tension in the fiber while a knot is tightened and then relaxed.

If cylindrical fibers are not required, a very interesting alternative to realize hemicylindrical CLCEs is to use direct ink write (DIW) 3D-printing of CLC oligomers. Sol et al. demonstrated a versatile method to print arbitrary CLCE patterns of multiple hemicylindrical filaments using direct ink writing.^49^ The pattern can be cross-linked into a connected 2D structure which can then be removed from the writing substrate, retaining its shape as a free-standing complex-shaped CLCE. The team also 3D-printed structurally colored actuators using a CLC oligomer as a precursor.^50^

## Conclusions and Outlook

I hope that the above examples serve to illustrate that polymerizable CLCs offer rich opportunities for innovative solutions to important current problems. The key feature that we utilize in all scenarios is the ability of CLCs to self-organize in a liquid state, which can be processed by standard fluidic methods, from blade coating and extrusion to microfluidic emulsification, as this allows us to easily prepare chiral selective Bragg reflectors in different desirable shapes and/or on different target substrates. Because the retroreflection color can easily be tuned and since the presence of reactive mesogens allows us to easily transfer the LC-derived structure into a glassy solid or a soft rubbery state, we have an incredibly versatile platform that opens diverse application opportunities.

While we have demonstrated all applications discussed here in relevant contexts in collaboration with experts in each application field, there are still some challenges ahead before commercial products are a reality. Most important is scale-up. We have recently replaced the cumbersome CSR production method in ref 15, requiring multiple time-consuming interruptions, by a fully continuous production method based on a commercial device for high-frequency production of multiple emulsions. We are currently fine-tuning the first prototype and will publish the result shortly. A similar scale-up problem is the main bottleneck for the CLCE fibers; filling of tubes is slow and allows only a few meters of fiber length at most. Together with collaborators specializing in industrially viable methods for spinning advanced fibers, we are exploring an interesting way to circumvent this bottleneck, which I hope will be mature for publication later this year.

Cost and durability are further challenges when we aim at large-scale application of CLC-based materials. Reactive mesogens are specialty chemicals that are significantly more expensive than monomers used for existing commodity polymers. While production cost will come down once enough customer interest exists to convince industrial producers to scale up synthesis, it will likely remain higher than for many simpler monomers. An interesting alternative is thus to start with naturally existing polymeric materials that form CLC phases; polysaccharides like cellulose and chitin here appear as prime candidates. If processing methods as simple and reliable as the thiol–acrylate-based click chemistry that currently dominates the LCE field can be made available using polysaccharide-derived precursors, a true revolution in applicability can be expected. A further diversification of the CLC chemistry will also be helpful in adapting materials to the varying demands of different use cases. The many ester groups of today’s dominating reactive mesogens raise concerns regarding long-term stability, of particular importance for outdoor applications as in SHM, and the thiol–acrylate bond has been shown to be sensitive to attack by hydrogen peroxide released by the immune system when LCEs are used as medical implants.^51^

To identify the most prolific application opportunities of CLCs, it is vital to interact with researchers, engineers, medical doctors, industry representatives etc., who are active in completely different fields, and get them excited about the possibilities. I would never have thought of using CLCEs for SHM without the discussions with Danièle, I did not even know about fiducial markers before Mathew introduced me to them, and I would never have been successful in utilizing the dynamic patterns of CSR arrays for secure authentication without the collaboration with Gabriele. Some of the most exciting opportunities for using LCs appear by sharing the excitement about these materials with people who have no prior knowledge of them, to whom all their properties are exotic.

With this history in mind, I end by encouraging every LC scientist (and others) to engage in discussions with researchers, business people, artists and many others who have complementary expertise and interests, sharing your passion with them, and learning about their challenges with curiosity. Such cross fertilization, in combination with research aiming to understand your topic at a deeper level, is a very powerful way of identifying both exciting new research fields and impactful application opportunities. That way, even old materials can experience new beginnings.
